# SPARC overexpression alters microRNA expression profiles involved in tumor progression

**DOI:** 10.18632/genesandcancer.130

**Published:** 2017-01

**Authors:** Bhavesh K. Ahir, Nasya M. Elias, Sajani S. Lakka

**Affiliations:** ^1^ Section of Hematology and Oncology, Department of Medicine, University of Illinois College of Medicine at Chicago, Chicago, IL, USA

**Keywords:** Medulloblastoma, SPARC, microRNA profiling, Tumor progression, Oncomine

## Abstract

Medulloblastoma is the most common malignant brain tumor in children. SPARC (secreted protein acidic and rich in cysteine), a multicellular non-structural glycoprotein is known to be involved in multiple processes in various cancers. Previously, we reported that SPARC expression significantly impairs medulloblastoma tumor growth *in vitro* and *in vivo* and also alters chemo sensitivity. MicroRNAs are a class of post-transcriptional gene regulators with critical functions in tumor progression. In addition, microRNA (miRNA) expression changes are also involved in chemo-resistance. Herein, we assessed microRNA (miRNA) profiling to identify the functional network and biological pathways altered in SPARC-overexpressed medulloblastoma cells. A total of 27 differentially expressed miRNAs were identified between the control and SPARC-overexpressed samples. Potential messenger RNA (mRNA) targets of the differentially expressed miRNA were identified using Ingenuity Pathway Analysis (IPA). Network-based functional analyses were performed on the available human protein interaction and miRNA-gene association data to highlight versatile miRNAs among the significantly deregulated miRNAs using the IPA, and the biological pathway analysis using the PANTHER web-based tool. We have identified six miRNAs (miR-125b1*, miR-146a-5p, miR-181a-5p, miR-204-5p, miR-219-5p and miR-509-3p) that are associated with SPARC sensitivity by comparison of miRNA expression patterns from the SPARC treated cells with the control cells. Furthermore, pathway enrichment analysis outline that these six microRNAs mainly belong to biological processes related to cancer related signaling pathways. Collectively, these studies have the potential to indicate novel biomarkers for treatment response and can also be applied to develop novel therapeutic treatment for medulloblastoma.

## INTRODUCTION

Medulloblastoma (MB) is the most common malignant brain tumor diagnosed in children that account for approximately 16-20 % of all pediatric brain tumors arising in the cerebellum [1–3]. Despite improved clinical outcomes with multimodal treatment including surgery, radiation and chemotherapy, tumor reappearance is frequent, and a 5-year survival rate of these patients is 60-70 % [4–7]. Furthermore, children diagnosed with medulloblastoma often suffer from cognitive and physical dysfunction resulting from current treatment of resection, followed by the long term toxicities associated with radiation and chemotherapy [8]. In addition, they have a tendency to disseminate throughout the central nervous system and identifying new molecular targets are required to improve outcomes.

SPARC also called osteonectin or basement membrane (BM 40) is a 32–35 kDa multifunctional collagen or calcium-binding ECM glycoprotein located at 5q33.1 and it consists of a single polypeptide with 285 amino acids including three biological structural domains; the acidic N-terminal (NT) domain, a follistatin-like domain and a Ca^2+^ binding extracellular domain [9–11]. It belongs to the matricellular family of secreted non-structural glycoproteins [12, 13]. In human carcinogenesis, SPARC plays remarkable roles in multiple biological functions including cell proliferation, cell cycle, adhesion, motility, apoptosis, extracellular (ECM) remodeling and on the activity of metalloproteases [14, 15]. Moreover, SPARC is also thought to play a critical role in differentiation of several cell types including epithelial differentiation [16], parietal endoderm differentiation, cardiomyogenesis in embryoid bodies [17], and differentiation of myoblasts [18]. SPARC plays an important role in neural and/or glial differentiation [19]. We have previously shown that SPARC expression caused neuronal differentiation [20] and caused autophagy mediated cell death [21]. Our studies also showed that SPARC enhances radiotherapy response in medulloblastoma *in vitro* and *in vivo* [22]. Understanding the role of signaling pathways involved in surviving and inducing cell death with SPARC expression is important for the development of more effective tumor therapies including SPARC gene therapy alone or in combination with other drugs [20, 23]. Several research groups have analyzed the mRNA expression profile of brain tumors to reveal novel gene markers for diagnosis and therapy and to better understand the regulatory pathways and genetic networks involved in medulloblastoma [24, 25].

MicroRNAs (miRNAs), an abundant class of ~22-nucleotide non-coding RNAs, regulate the expression of genes at post transcriptional level [26]. It has become increasingly evident that miRNA plays an essential role in the brain including neurogenesis and differentiation [27, 28]. To date, miRNAs are thought to regulate almost 60 % of all protein-coding genes in humans and participate in the regulation of almost every cellular processes investigated [29]. Recent studies have shown that approximately 60-70 % of the currently identified miRNAs are expressed in the brain [30, 31]. The miRNA-related genetic alterations are implicated in multiple human diseases and pathological processes including cancer [32]. MicroRNAs not only act as oncogenes by inhibiting translation of tumor suppressor mRNAs, but they can also act as tumor suppressor genes as well, by inhibiting translation of oncogenic mRNAs [26, 33, 34]. Several groups of miRNAs have been identified to regulate the expression of tumor-associated genes [35, 36], while others seem to hold prognostic value in predicting the survival of cancer patients [37].

In this study, we identified the miRNA expression profile in SPARC overexpressed human medulloblastoma cells using microarray technology. Using bioinformatics tools and systems biology approach, we systematically identified differentially expressed miRNAs, miRNA-mRNA predictive targets, biological functions and disease signature, network analysis and the biological signaling pathways of gene targeted by these SPARC modulated differentially expressed miRNAs. We further selectively confirmed six miRNAs with statistically significant differential expression by miRNA quantitative real-time polymerase chain reaction (qRT-PCR) in SPARC expressed cells and identified that the expression of these six miRNA targets are altered in human medulloblastoma patient samples.

## RESULTS

### SPARC expression in medulloblastoma cells

**Figure 1:**
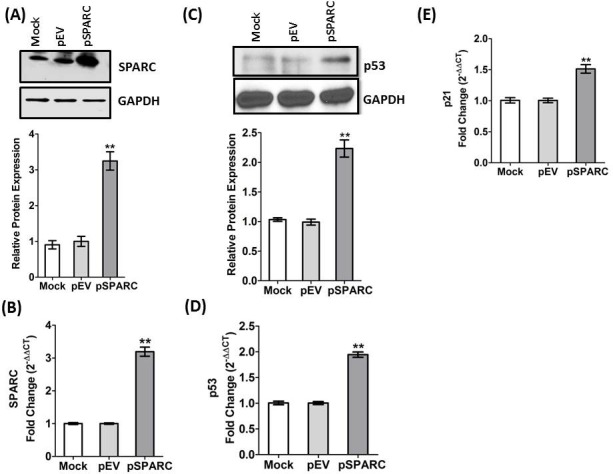
Expression of SPARC in D283 medulloblastoma cells Medulloblastoma cells were transfected with plasmid containing full-length SPARC cDNA (pSPARC) or empty vehicle control (pEV) or mock (untreated) control for 36 hrs. (A) SPARC protein levels were determined in total cell lysates by western blotting analysis using SPARC specific primary antibody. GAPDH was used to confirm equal loading. (B) Total RNA was extracted using Trizol reagent, and qRT-PCR was performed for SPARC mRNA transcript level. (C) p53 protein levels were determined in total cell lysates by western blotting analysis using p53 specific primary antibody. GAPDH was used to confirm equal loading. (D) Total RNA was extracted using Trizol reagent, and qRT-PCR was performed for p53 mRNA transcript level or (E) p21 mRNA transcript level. Total protein levels were quantified by densitometric analysis as shown in the corresponding bar graph. **P < 0.01 compared to the mock control or pEV control group (mean ± SE, *n* = 4).

We previously demonstrated that human medulloblastoma tissue samples expressed very low or minimal levels of SPARC when compared with normal cerebellum [20]. Furthermore, we also showed that SPARC protein and mRNA levels were increased up to 3–4-folds in D425 and UW228 medulloblastoma cell lines transfected at a 2 μg/ml of pSPARC compared to controls [23]. Herein, we sub-cloned human SPARC full-length cDNA into a pcDNA3.1 mammalian expression vector (pSPARC). We transfected medulloblastoma D283 cells with pSPARC for 36 h. Total protein and total RNA was isolated from the cells at 36 h after post-transfection, and immune blot analysis and qRT-PCR was performed to detect SPARC expression levels in medulloblastoma cells. We found more than 3-fold increase in relative SPARC protein and mRNA transcript expression in D283 medulloblastoma cells as compared to parental (mock) and empty vector (pEV) controls (*p < 0.01*; Figure 1A and 1B). Our previous studies also indicated that the Sub-G1 region corresponding to apoptotic cells was increased by 56.7 ± 4% in SPARC overexpressed D283 cells as compared to 5.0 ± 2% in empty vector treated controls. In. addition, SPARC significantly increased TUNEL-positive apoptotic cells in D283 medulloblastoma cells in a concentration dependent manner. Further, we also demonstrated that SPARC overexpression increased caspase-3 and caspase-8 activities and enhanced PARP cleavage when compared to mock or an empty vector controls in D283 medulloblastoma cells [20, 21]. Fluorescence activated cell sorting (FACS) analysis indicated that SPARC expressed cells were arrested in G2/M phase compared to mock or empty vector treated controls in medulloblastoma cells [23]. We therefore explored potential mechanisms of the altered cell cycle arrest profile. We determined p53 and p21 level in SPARC overexpressed cells by immunoblotting and qRT-PCR analysis. SPARC expression induced p53 and p21 levels by 2–3 fold compared to controls (Figure 1C, D and E). These studies suggest that p53/p21^(WAF1/CIP1)^ pathway is a critical component of a cell cycle regulatory pathway(s) that controls the occurrence of G2M arrest in SPARC expressed D283 medulloblastoma cells.

### Medulloblastoma miRNA profiles identified putative candidate miRNA markers

**Figure 2:**
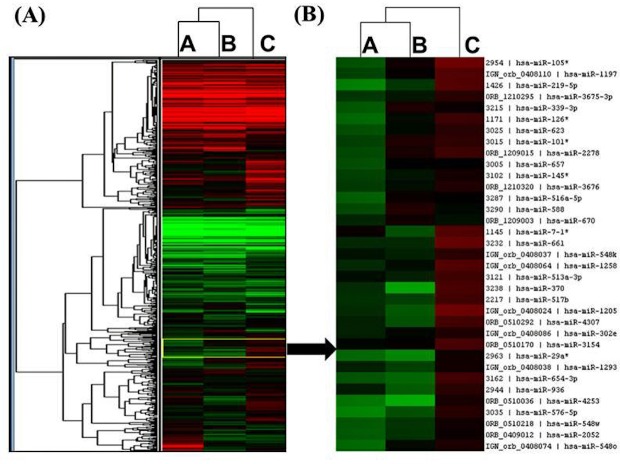
miRNA profiling of SPARC overexpressed or SPARC underexpressed medulloblastoma cells (A) The unsupervised hierarchical clustering of the 729 miRNAs with acceptable detection intensities using Gene Cluster 3.0 Software. (B) An enlarged view of a representative section of the clustered image representing the subset of miRNAs from the unsupervised hierarchical clustering of the 729 miRNAs. Heatmap color scale represents fold increase (Red) or decrease (Green) from baseline. A: pEV (empty vehicle) control samples, B: SPARC-overexpressed samples, C: SPARC-underexpressed samples.

We used custom multi-species microarrays containing 1209 probes covering 1220 human mature miRNAs, to detect differentially expressed miRNAs in SPARC overexpressed D283 medulloblastoma cells. The custom microarray chip containing 1209 probes covering 1220 human miRNAs present in the miRBase version 16.0 database released in 2010 [38]. Figure 2A shows the unsupervised hierarchical clustering of the 729 miRNAs with acceptable detection intensities. A very remarkably different clustering pattern was observed in SPARC overexpressed versus SPARC under expressed medulloblastoma samples compared to parental/control medulloblastoma samples (Figure 2A and B).

**Figure 3:**
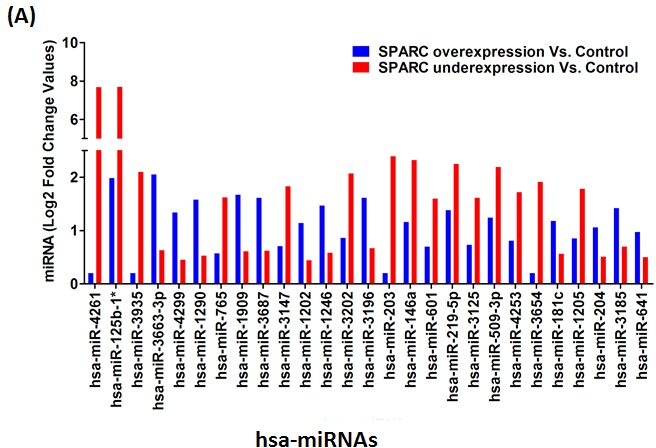

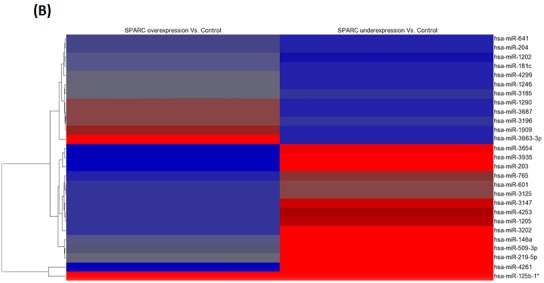
SPARC regulated differentially expressed miRNAs in medulloblastoma cells (A) Shows a graphical view representation of Log2 fold change in miRNA level between control (pEV) samples versus SPARC overexpressed and/or SPARC underexpressed medulloblastoma cells. Abbreviation: hsa miRNAs: human microRNAs. (B) Represents a hierarchical clustering of SPARC modulated differentially expressed 27 miRNAs. Blue color indicates relative low expression, red color indicates relative high expression, and gray color indicates no changes in the expression patterns.

To compile a set of targeted miRNAs for further investigation and confirmation, the list of 623 Log2 transformed and normalized raw spot intensities were examined for difference between the group by at least 2-fold differences between the control and SPARC overexpression and/or SPARC underexpression (Supplementary Material, Table S1). A total of 27 differentially expressed probes exhibited at-least two-fold difference between the control and SPARC overexpression or SPARC-underexpression samples (Figure 3A). Hierarchical unsupervised clustering of medulloblastoma using the most significantly differentially expressed miRNAs is represented in a heat map (Figure 3B). A set of 27 differentially expressed miRNAs have been clustered by unsupervised hierarchical clustering analysis, generating a dendogram that shows a clear separation of SPARC overexpression versus control and SPARC underexpression versus control (Figure 3B).

**Table 1:** Primers used in quantitative reverse transcriptase-polymerase chain reaction (qRT-PCR) analysis of miRNAs

MicroRNA	Primer Sequence (52-32)
has-miR-204-5p	GCC AGA TCT GGA AGA AGA TGG TGG TTA GT
has-miR-219-5p	TGA TTG TCC AAA CGC AAT TCT
has-miR-509-3p	GTC TGA TTG GTA CGT CTG
has-miR-146a-5p	GGCGATGAGAACTGAATTCCA
has-miR-181a-5p	GAA CAT TCA ACG CTG TCG GTG A
has-miR-125b-1*	TCC CTG AGA CCC TAA CTT GTG

Abbreviations: has-human, miR-microRNA

### Prediction of transcriptional targets of differentially expressed miRNAs using IPA

We performed *in silico* analysis to understand genomic changes likely regulated by this set of 27 differentially expressed miRNAs. Using the Ingenuity miRNA Target Filter, based on knowledgebase of predicted and experimentally observed miRNA-mRNA relationships, we identified 1,076 mRNA that were experimentally or predicted targets of the 15 miRNAs. Inclusion of only the experimentally observed downstream mRNA targets resulted in a set of 6 miRNAs with 82 target mRNA, of which 81 were unique (Table 2; Supplementary Material, Table S2). This *in silico* analysis predicted miR- 125b-1* to target 3 mRNAs, miR-146a-5p to target 47 mRNAs, miR-181a-5p to target 14 mRNAs, miR-204-5p to target 12 mRNAs, miR-219a-5p to target 5 mRNAs, and miR-509-3p to target 1 mRNAs for a total 82 targets (see Table 2).

**Table 2:** Six miRNAs show putative miRNA-mRNA relationships using the IPA miRNA Target Filter analysis, based on a knowledge base of predicted and experimentally observed relationships

ID	miRNA Symbol	IPA Experimentally Observed mRNA Targets
has-miR-125b-1*	miR-125b-1-3p (miRNAs w/seed CGGGUUA)	3
has-miR-146a	miR-146a-5p (and other miRNAs w/seed GAGAACU)	47
has-miR-181c	miR-181a-5p (and other miRNAs w/seed ACAUUCA)	14
has-miR-204	miR-204-5p (and other miRNAs w/seed UCCCUUU)	12
has-miR-219-5p	miR-219a-5p (and other miRNAs w/seed GAUUGUC)	5
has-miR-509-3p	miR-509-3p (miRNAs w/seed GAUUGGU)	1

### Network and functional enrichment of SPARC overexpression mediated miRNA targets

**Figure 4:**
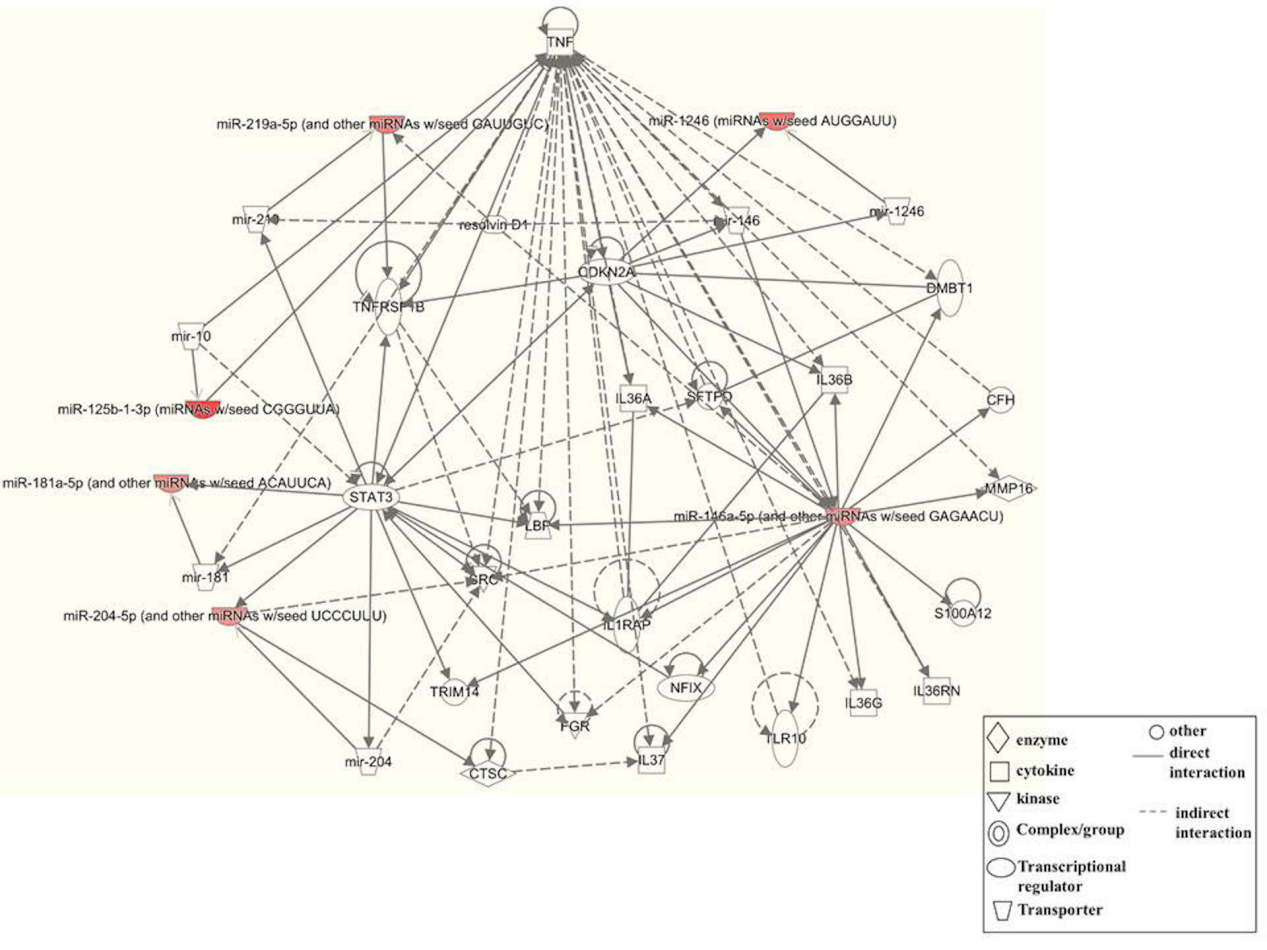
Molecular networks identified by Ingenuity Pathway Analysis (IPA) The most significant molecular network by IPA pathway enrichment analysis in SPARC overexpressed cells. Red symbol represents miRNAs targets and clear symbol is associated with protein targets The IPA proprietary database manually curates information about gene-phenotype associations, molecular interactions, regulatory events, and chemical knowledge to provide a global molecular network. Related network was algorithmically constructed based on connectivity, as enabled through IPA. Statistical significance of each biological function in each resulting network was calculated based on Fisher’s exact test with *p < 0.05* considered as significant.

Functional enrichment analysis of microRNAs and their target genes was performed to identify unique, similar and common sets of genes/proteins. We constructed molecular networks with the 81 unique mRNAs predicted to be regulated by 6 differentially expressed miRNAs in SPARC expressed medulloblastoma cells using the IPA. Fig. 4B indicates that the most significantly enriched network were associated with inflammatory cytokines (*p < 10^−10^*) in SPARC over expressed cells. Tumor necrosis factor (TNF) was identified as a key node in these interactions. The top most 10 significantly associated networks were inflammatory disease, inflammatory response to immune cell, cellular movement and immune cell trafficking, cellular development including cellular growth and proliferation, cell morphology, hematological system development and function, cell-to-cell signaling, cell cycle and inflammatory response to neurological disease (Table 3; Supplementary Material, Table S3).

**Table 3:** Ten most significant biological functions and disease signatures associated with SPARC-overexpressed medulloblastoma cells compare to controls

Biological Function/Disease Signature	p-Value
Inflammatory Diseases	9.72E-09
Inflammatory Response	2.75E-08
Cellular Movement, Immune Cell Trafficking	8.23E-08
Cellular Development, Cellular Growth and Proliferation	1.23E-07
Cell Morphology, Hematological System Development and Function, Inflammatory Response	1.40E-07
Cell-To-Cell Signaling and Interaction, Hematological System Development and Function, Immune Cell Trafficking, Inflammatory Response	2.65E-07
Cell-To-Cell Signaling and Interaction	3.75E-07
Cell Cycle	4.70E-07
Inflammatory Response, Neurological Disease	4.72E-07
Cell-To-Cell Signaling and Interaction, Inflammatory Response	6.06E-07

### Pathway analysis of differentially expressed miRNAs

We performed pathway analysis using PANTHER database in order to understand which signaling pathways are altered with SPARC expression in medulloblastoma cells. The 81 unique mRNA gene list was entered into the PANTHER database in order to identify significantly over-and under-represented pathways, molecular functions and biological processes, in comparison with a reference gene (Homo *sapiens* Whole Genome list) (Table 4). From the initial list of 81 unique genes, PANTHER was unable to map 3 mRNAs. For this reason, all analyses were performed on a total 78 gene targets. Table 4 shows statistically significant (*p < 0.05*) pathways, molecular function classes and biological process relevant to the 78 gene targets genes. In all cases, a statistically significant (*p < 0.05*) overrepresentation of the number of target genes per category was also observed. A total of 6 biological signaling pathways were significantly associated with SPARC mediated expression in medulloblastoma cells (*p < 0.05*) (Table 4). The top significant pathways associated with SPARC mediated expression in medulloblastoma cells include toll receptor signaling pathway, interleukin signaling pathway, apoptosis signaling pathway, angiogenesis signaling pathway, inflammation mediated chemokine and cytokine signaling pathway and platelet derived growth factor (PDGF) signaling pathway.

**Table 4:** Biological pathway analysis using the PANTHER web tool. PANTHER over-representation analysis of target genes list

PANTHER CLASSIFICATION CATEGORY	Number of genes			Over−/Under-represented (+/−)	p-Value	% of target list^d^
Pathways	Reference list^a^	Target list^b^	Expected^c^			
Toll receptor signaling pathway	56	8	0.21	+	8.39E-09	10.27
Interleukin signaling pathway	97	7	0.36	+	1.45E-05	8.97
Apoptosis signaling pathway	115	6	0.43	+	8.00E-04	7.69
Angiogenesis Signaling Pathway	154	6	0.58	+	4.11E-03	7.69
Inflammation mediated by chemokine and cytokine signaling pathway	245	9	0.92	+	5.86E-05	11.54
PDGF signaling pathway	138	5	0.52	+	2.79E-02	6.11

Pathways, molecular function classes and biological processes resulted significantly over or under represented by 78 mRNA targets.

a Number of genes in the reference list that map to this PANTHER classification category.

b Number of genes in the target genes list that map to this PANTHER classification category.

c c-Expected value is the number of genes that could be expected in target gene list for this PANTHER category based on the reference list.

d d-Percentage of genes (%) in the target list out of the total considered genes in PANTHER (n=78 mRNA targets). *p-Values* are determined by binominal statistical analysis with Bonferroni Correction: a *p < 0.05* was considered significant.

### Analysis of putative microRNA targets in medulloblastoma patient samples

To further investigate the biological relevance of the six miRNAs differentially regulated in SPARC expressed cells, we looked for the known targets of these six miRNAs in medulloblastoma patient samples (Table 5). The analysis was focused on the research of targets experimentally validated in previously published studies using publicly available human medulloblastoma datasets within the Oncomine Database (www.oncomine.org/) [39, 40]. We mined 81 mRNA targets of the six differentially expressed miRNAs in desmoplastic medulloblastoma cancer type samples versus normal control samples (n=4 normal brain cerebellum control samples and n=14 desmoplastic patient samples). In all cases, a statistically significant altered expression of the number of target genes was observed in medulloblastoma patient samples (see Table 5 and Supplementary Material, Table S2).

**Table 5:** The fold change values of putative gene targets of SPARC modulated differentially expressed six miRNAs in desmoplastic medulloblastoma patient’s samples versus normal control samples, from the data-mining platform, Oncomine Database

MicroRNA (miRNA)	Gene Targets	Fold change
has-miR-125b-1*	TNF	−1.15
has-miR-125b-1*	IL1B	−4.59
has-miR-125b-1*	IL13	−1.50
has-miR-219-5p	TNFRSF1B	−1.54
has-miR-219-5p	PLCG2	27.2
has-miR-219-5p	ENPP6	ND
has-miR-219-5p	CD14	4.0
has-miR-219-5p	ALOX5	1.19
has-miR-509-3p	NTRK3	170
has-miR-181c	TRA	−1.4
has-miR-181c	TIMP3	2.52
has-miR-181c	TCL1A	1.61
has-miR-181c	NOTCH4	1.15
has-miR-181c	NLK	−1.73
has-miR-181c	KRAS	1.10
has-miR-181c	GRIA2	−1.52
has-miR-181c	GATA6	7.59
has-miR-181c	ESR1	1.89
has-miR-181c	CDX2	32.95
has-miR-181c	CDKN1B	2.35
has-miR-181c	CD69	1.75
has-miR-181c	BCL2	1.55
has-miR-181c	AICDA	ND
has-miR-146a	TRAF6	1.75
has-miR-146a	TLR9	ND
has-miR-146a	TLR4	1.31
has-miR-146a	TLR10	ND
has-miR-146a	STAT1	2.18
has-miR-146a	PTGES2	ND
has-miR-146a	PLEKHA4	ND
has-miR-146a	PA2G4	ND
has-miR-146a	NOS2	ND
has-miR-146a	NFIX	25.8
has-miR-146a	MR1	−1.21
has-miR-146a	MMP16	−2.35
has-miR-146a	LTB	1.89
has-miR-146a	LBP	ND
has-miR-146a	IRAK2	ND
has-miR-146a	IRAK1	2.96
has-miR-146a	IL37	ND
has-miR-146a	IL36RN	ND
has-miR-146a	IL36G	ND
has-miR-146a	IL36B	ND
has-miR-146a	IL36A	ND
has-miR-146a	IL1RL2	−2.0
has-miR-146a	IL1RAPL2	ND
has-miR-146a	IL1RAP	ND
has-miR-146a	IL1R1	−2.5
has-miR-146a	IL1F10	ND
has-miR-146a	IL12RB2	5.0
has-miR-146a	IL10	−2.92
has-miR-146a	IFNB1	−16.0
has-miR-146a	IFNA1/IFNA13	1.89
has-miR-146a	FADD	2.44
has-miR-146a	CXCR4	103
has-miR-146a	CXCL8	7.87
has-miR-146a	CRP	−1.56
has-miR-146a	COL13A1	−60.0
has-miR-146a	CHUK	ND
has-miR-146a	CFH	ND
has-miR-146a	CDKN3	−1.26
has-miR-146a	CD40	−4.0
has-miR-146a	CD1D	7.78
has-miR-146a	CCR3	−2.38
has-miR-146a	CCNA2	9.89
has-miR-146a	CCL8	−5.56
has-miR-146a	CAMP	2.40
has-miR-146a	C8A	1.17
has-miR-146a	BRCA1	5.29
has-miR-204	SOX4	56.2
has-miR-204	SHC1	12.7
has-miR-204	MMP9	−1.04
has-miR-204	MMP3	2.79
has-miR-204	ITGB4	2.46
has-miR-204	HMGA2	21.7
has-miR-204	EFNB1	−3.4
has-miR-204	CDH11	14.7
has-miR-204	CDC25B	−3.8
has-miR-204	BMP1	5.70
has-miR-204	ATP2B1	−2.14
has-miR-204	ARPC1B	1.89

[Abbreviation: ND: not detected]

### Expression of SPARC modulated miRNAs in medulloblastoma

**Figure 5:**
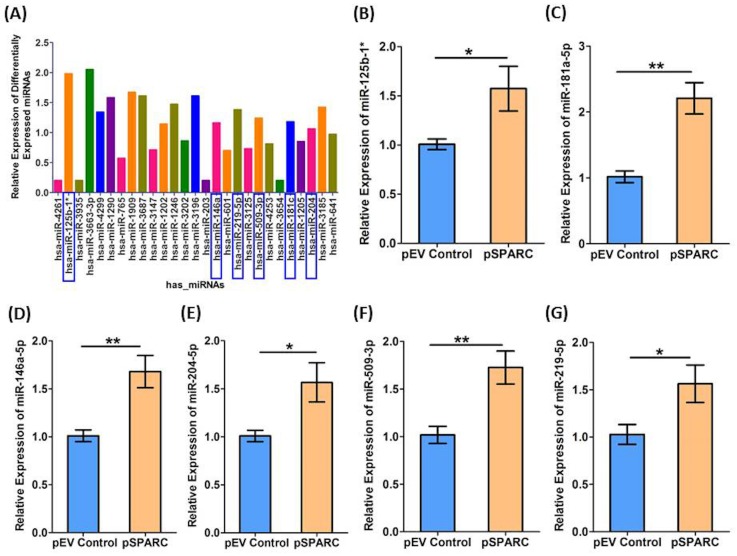
Validation of miR-125-b1*, miR-181a-5p, miR-146a-5p, miR-204-5p, miR-219-5p and miR-509-3p upregulation by quantitative real time PCR (qRT-PCR) (A) A bar graph showing 27 diffentially expressed miRNAs in SPARC overexpressed medulloblastoma cells versus pEV controls of which 6 miRNAs were selected (represented and highlighted in blue color square box in graph) based on *in silico* prediction of miRNA-mRNA prediction using the IPA. (B-G) Relative expression of has-miR-125b- 1*(*p < 0.05), has-miR-181a-5p (**p < 0.001), has-miR-146a-5p, (**p < 0.001), has-miR-204-5p (*p < 0.05), has-miR-509-3p (*p < 0.001), and has-miR-219-5p (*p < 0.05) in SPARC overexpressed medulloblastoma samples (pSPARC) compared to empty vector (pEV) control group.

Relative changes in miRNA expression observed in microarray analysis were validated using qRT-PCR. We evaluated the 6 miRNAs identified based on *in silico* prediction for experimentally observed miRNA-mRNA target predictions using the IPA in SPARC overexpressed cells (Figure 5A). qRT-PCR was performed on miR-125b-1* (FC = 1.57), miR-181a-5p (FC = 2.21), miR-146a-5p (FC = 1.68), miR-204-5p (FC = 1.57), miR-509-3p (FC = 1.73) and miR-219-5p (FC = 1.57) in SPARC overexpressed medulloblastoma cells (pSPARC) compared to empty vehicle (pEV) treated control samples. There was a significant increase in relative fold change of all the six microRNAs (Figure 5B-G). We next evaluated the role of these six microRNAs in other disease states along with their functional relationships as summarized in Table 6. The top significant pathways associated with these microRNAs that are regulated by these six microRNAs include cell proliferation, growth, invasion, angiogenesis and apoptosis.

**Table 6:** Expression and functional relationships of identified six miRNAs and their role in other disease states

miRNA	Direction of Deregulation	Other Disease States	Functional Assay	Reference
	Up	Leukemia	Regulator of apoptosis	[79]
hsa-miR125-b1	Up	Gastric cancer	Pro-proliferative, anti-apoptotic	[80]
	Down	Bladder cancer	Cell migration and invasion	[81]
	Down	Breast cancer	Cell migration and invasion	[50]
	Down	Breast cancer	Cell proliferation and apoptosis	[82]
	Down	Endometrial cancer	Cell invasion	[83]
	Down	Hepatocellular carcinoma (HCC)	Regulator of apoptosis	[84]
	Down	Hepatocellular carcinoma (HCC)	Cell invasion and angiogenesis	[85]
	Down	Osteosarcoma	Cell proliferation and cell migration	[86]
	Down	Ovarian cancer	Cell proliferation and angiogenesis	[87]
	Down	Skin cancer	Cell proliferation	[88]
	Up	Glioblastoma	Regulator of apoptosis	[46]
	Up	Glioblastoma	Cell proliferative and anti-apoptotic	[44]
	Down	Glioblastoma	Regulator of angiogenesis	[49]
	Up	Prostate cancer	Pro-proliferative and anti-apoptotic	[89]
hsa-miR-146a-5p	Up	Breast cancer	Cell migration and invasion	[56]
	Up	Papillary thyroid carcinoma	Cell proliferations and growth	[90]
	Up	Cervical cancer	Cell growth	[91]
	Up	Pancreatic cancer	Cell invasion	[57]
	Down	Inflammatory diseases	Cell death	[92]
	Down	Non-small cell lung carcinoma	Cell growth, migration and apoptosis	[93]
hsa-miR-181a-5p	Down	Prostate cancer	Cell growth, motility and invasiveness	[94]
	Down	Gastric cancer	Cell proliferation, invasion and cell cycle	[95]
	Down	Breast cancer	Cell migration and angiogenesis	[96]
	Down	Colon cancer	Cell migration, invasion and angiogenesis	[96]
	Down	Glioma cancer	Cell proliferation, invasion and apoptosis	[60]
	Down	Glioma cancer	Cell invasion, proliferation and apoptosis	[97]
hsa-miR-204-5p	Down	Colorectal cancer	Cell proliferation and apoptosis	[98]
	Down	Gastric cancer	Cell invasion and apoptosis	[99]
	Down	Endometrial cancer	Cell growth, migration and invasion	[64]
hsa-miR-219-5p	Down	Hepatocellular carcinoma	Cell proliferation and cell cycle arrest	[100]
	Down	Papillary thyroid cancer	Cell proliferation, migration and apoptosis	[101]
has-miR-509-3p	Down	Ovarian cancer	Cell proliferation, migration and invasion	[102]
	Up	Renal cell carcinoma	Cell migration and apoptosis	[72]

## DISCUSSION

MicroRNA signature can predict the prognosis and therapy response for appropriate therapeutic management. Our previous studies have shown that SPARC expression was very low in human medulloblastoma tissue samples when compared with normal cerebellum tissue samples [20]. We also showed that SPARC expression inhibits medulloblastoma tumor growth in an intracranial mouse model [21, 23]. We also observed that SPARC expression induced the expression of neuronal marker genes (such as MAP-2, NeuN, nestin and neurofilament) [20]. Further, we also showed that SPARC expression enhanced radio response and combined treatment of SPARC and irradiation resulted in increased cell death when compared to cells treated with irradiation alone *in vitro* and *in vivo*. SPARC expression suppressed irradiation-induced checkpoints-1,-2 and p53 phosphorylation and DNA repair gene, XRCC1. In addition, SPARC expression suppressed irradiation induced SOX-4 mediated DNA repair [22]. Further, we also showed that SPARC altered cisplatin sensitivity by modulating the Let-7f-1 miRNA/ HMGB1 axis in medulloblastoma cells. In this study, we demonstrated that autophagy was involved in SPARC expression mediated resistance to cisplatin. Further, SPARC expression suppressed miR-let-7f-1 expression which resulted in disrupted repression of High Mobility Group Box 1 (HMGB1), a critical regulator of autophagy [41]. MicroRNAs can act as oncogenes or tumor suppressors and are capable of modulating several targets in multiple genetic pathways. Identification of a specific pattern of expression of miRNAs in SPARC expressed cells could shed light on the underlying mechanisms of SPARC mediated tumor suppressive and chemosensitising effects. We therefore performed global expression analysis for microRNAs using miRNA microarray to explore the miRNAome in SPARC expressed medulloblastoma and control cells. Twenty-seven miRNAs were identified as differentially expressed between SPARC expressed cells compared to control cells. *In silico* analysis using IPA analysis based on the research of targets experimentally validated in previously published studies indicated that miR-125b-1*, miR-146a-5p, miR-181a-5p, miR-204-5p, miR-509-3p and miR-219-5p were upregulated in SPARC expressed cells. The interactive networks and hubs revealed functional cooperatively of these six miRNAs and target groups in the various signaling pathways. The functional and pathway enrichment analysis identified that different oncogenic pathways regulated by these six microRNAs were previously demonstrated to be involved in cancer progression.

Our studies show that miR-125b was increased with SPARC expression. This is consistent with a recent study in which they showed that miR-125b was upregulated in medulloblastoma [42]. miR-125b is the most abundant miRNA in the brain and its play an important role in embryonic brain development including neural development by repressing multiple targets [43]. Furthermore, miR-125b on one hand promotes proliferation and growth of glial and neuroblastoma tumor cells *in vivo* and *in vitro* [44–46] and on the other hand, it behaves as a tumor suppressor in glioblastoma-associated endothelial cells, glioma stem cells and medulloblastoma [47–49]. Several studies also indicate that miR-125b functions as a tumor suppressor. The expression of miR-125b is downregulated in various human cancers including glioblastoma, prostate cancer, ovarian cancer, and breast cancer by suppressing oncogenes such as EST1, ERBB2, ERBB3, and BAK1 as its targets [50, 51]. Another study also demonstrated that silencing of miR-125b1 leads to the activation of the ETS1 proto-oncogene resulting in worse prognosis in breast cancer patients [51]. Further miR-125b was implicated in neuronal differentiation of mouse P19 embryonal carcinoma cells [52]. The depletion of miR-125b suppressed the proliferation of differentiated human neuroblastoma cells *in vitro* [53]. This is consistent with our previous studies demonstrating that SPARC expression suppressed proliferation and induced neuronal differentiation in medulloblastoma cells [20, 23] suggesting that mir-125b could be a key player in SPARC mediated tumor suppressive effects in medulloblastoma cells.

Our data also indicates that SPARC expression enhanced miR-146a expression. This result is consistent with a demonstrated role of miR-146a as a tumor suppressor in several studies. Upregulation of miR-146a plays an inhibitory role in restricting the formation of glioma stem-like cells and tumor burden [54]. Overexpression of miR-146a inhibits the proliferation and survival of breast, prostate, and pancreatic cancer cells through the downregulation of its targets including ROCK1, EGFR, and MTA-2 [55–58]. Deletion of miR-146a spontaneously developed subcutaneous flank tumors in mice [59]. Ectopic expression of miR-146a inhibits tumor development of a human glioblastoma cell line in an orthotropic xenograft model by downregulation of Notch1, which plays a key role in neural stem cell maintenance and is a direct target of miR-146a [54]. Conversely, knockdown of miR-146a by microRNA sponge upregulates Notch1 and promotes tumorigenesis of malignant astrocytes [54]. We have previously demonstrated that SPARC stimulates neuronal differentiation of medulloblastoma cells by suppressing Notch signaling [20]. Our present study demonstrates that SPARC expression upregulated miR- 146a as determined by quantitative real time PCR analysis suggesting a possible role of miR-146a in SPARC induced neuronal differentiation.

miR-181 expression was also enhanced in SPARC expressed medulloblastoma cells compared to controls. Several studies have showed that mir-181a and mir-181b act as tumor suppressor genes. mir-181a/b was down- regulated in gliomas and re-introduction of these miRNAs on glioma cell lines results in cell growth inhibition, suppression of invasiveness and induction of apoptosis [60]. miR-181 expression was shown to enhance radio and chemo sensitivity [61–63]. We have previously shown that SPARC enhances radio response in medulloblastoma [22]. Taken together, these findings support the hypothesis that mir-181 may be involved in enhancing radio response in medulloblastoma.

We also demonstrate increased expression of miR-204 in SPARC overexpressed medulloblastoma cells. Several studies have also shown that miR-204-5p is frequently downregulated in papillary thyroid carcinoma, gastric cancer, colorectal cancer, neuroblastoma and endometrial carcinoma, suggesting a common role of miR- 204-5p in human tumorigenesis [64–68]. Downregulation of miR-204-5p in human gliomas is correlated with poor patient prognosis, and overexpression of miR-204-5p inhibits the proliferation, migration and invasiveness of glioma cells *in vitro* and *in vivo* by directly targeting RAB22A (a member of the RAS oncogene family) [67]. Further, miR-204 was shown to regulate autophagy in renal clear cell carcinoma (RCC) by regulating the expression of LC3 [69]. Our previous studies demonstrated that SPARC expression induced autophagy by modulating LC3 [20, 21]. However, it remains to be determined whether mir-204 induces autophagy in SPARC mediated autophagy in medulloblastoma cells.

Our studies also show that miR-219 is upregulated with SPARC expression. Previous studies demonstrate that miR-219 is downregulated in medulloblastoma tumors and re-introduction of miR-219 inhibits proliferation and suppresses the invasiveness of D283-medulloblastoma cells [70]. Previous data, in combination with our current findings, raise the possibility that induction of miR-219-5p expression may contribute to SPARC mediated suppression of cell proliferation, migration and invasion [20, 22, 23].

Our study also observed that another miR-509- 3p associated with cell proliferation and migration is upregulated in SPARC overexpressed cells. miR-509-3p, a significant regulator of the MAP3K8 oncogene was shown to be down regulated in tumor cells [71]. Furthermore, downregulation of miR-509-3p was shown to be a tumor suppressor in tumor cells and associated with tumor cell invasion and migration [72].

We also analyzed the experimentally validated targets of these six miRNAs. Among the validated targets we found transcription factors, cytokines, differentiation, oncogenes, genes involved in histone deacetylation and in cancer cell invasion, apoptosis and angiogenesis. Concurrent with the divergent roles of SPARC in carcinogenesis [15], our study also observed that SPARC altered the expression levels of microRNAs that regulate several signaling pathways involved in tumor progression including toll receptor signaling pathway, interleukin signaling pathway, apoptosis signaling pathway, angiogenesis signaling pathway, inflammation mediated chemokine and cytokine signaling pathway and platelet derived growth factor (PDGF) signaling pathway. Taken together, this established a clear mechanistic interaction underlying miRNA deregulation and tumorigenesis in medulloblastoma cells. Further investigation including functional and mechanistic studies must be undertaken in order to address the biological implications of these miRNAs and to elucidate their involvement in SPARC mediated effects on tumor suppression, treatment response and resistance.

In conclusion, we established that SPARC expression modulated microRNA profile in medulloblastoma cells. Several of the identified miRNAs are involved in tumor progression, suggesting that their deregulated expression may be involved in medulloblastoma tumorigenesis. Studying the targets of deregulated miRNAs genes may elucidate biological functions involved in medulloblastoma pathogenesis.

## MATERIALS AND METHODS

### Cells and culture condition

Human medulloblastoma cell line D283 was purchased from American Type Culture Collection (ATCC) for this study. D283 cells were authenticated by DNA profile, using the short tandem repeat (STR), cytogenetic analysis, and isoenzymes obtained from ATCC. To obtain consistent results, cells were frozen at third or fourth passage. D283 medulloblastoma cells were cultured in Improved MEM (Zn tonic option medium without phenol red) supplemented with 10 % FBS, 100 units/ml penicillin and 100 μg/ml streptomycin (Gibco BRL, Life Technologies, NY) and collected for use as indicated. Medulloblastoma cells were maintained in a humidified incubator containing 5 % CO_2_ at 37°C.

### Construction of pSPARC and cell transfection

An 1100 bp cDNA human SPARC was amplified by reverse transcription PCR using synthetic primers and cloned into a pcDNA3.1 vector (Invitrogen, San Diego, CA) in sense orientation as described previously [20–23]. Cells were transfected with pcDNA3.1 plasmid containing full length cDNA of SPARC (pSPARC) or empty vector (pEV, a pcDNA vector carrying no vector) using FuGene^®^HD (Roche, Indianapolis, IN) as per manufacturer’s instructions. For the transfections, cells were cultured in culture flasks (D283) until cells were reached 60-80 % confluence. 7 μg of plasmid DNA were mixed with FuGene^®^HD in serum free medium per manufacturer’s instructions, and 30 minutes later added to the cells. After allowing 8 hour (h) for optimal transfection, the serum free medium was replaced by complete, supplemented medium, and cells were incubated for 36 h. Cells were harvested for the collection of total RNA and whole cell lysate (total protein) for miRNA profiling with qRT-PCR validation and Western blotting analysis respectively.

### Western Blotting Analysis

Medulloblastoma (D283) cells were transfected with mock, pEV or pSPARC for 8 h, and then cells were cells were collected at 36 h post-transfection and lysed in RIPA lysis buffer (50 mM/ml Tris-HCL pH 8.0, 150 mM/ml NaCl, 1 % sodium deoxycholate, 0.1 % SDS) containing 1 mM sodium orthovanadate, 0.5 mM PMSF, 10 mg/ ml leupeptin. The protein concentration was determined by Bradford assay [73]. Equal amounts of protein were resolved on SDS-PAGE and then subject to western transfer onto nitrocellulose membranes and kept overnight. The blots were blocked with 5 % nonfat dry milk in TBST (Tris buffer saline with 0.1 % Tween-20) and probed overnight with goat anti-rabbit SPARC polyclonal primary antibody (1:1000; Santa Cruz, CA, USA) and mouse monoclonal p53 primary antibody (1:500; Santa Cruz, CA, USA), followed by incubation with species-specific HRP-conjugated secondary antibodies. An ECL system was used to detect chemiluminescent signals. GAPDH antibody (1:10000; Santa Cruz, CA, USA) was used to verify equal amounts of proteins were loaded in all lanes.

### MicroRNA (miRNA) microarray and analysis

Total RNA was extracted from D283 cells using TRIZOL reagent (Invitrogen, Carlsbad, CA) as per standard control protocol. Quality control of the total RNA samples was assessed using UV Spectrophotometry and agarose gel electrophoresis. The samples were DNase digested and low-molecular weight (LMW) RNA was isolated by ultrafiltration through YM-100 columns (Millipore) and subsequent purification using the RNeasy MinElute Clean-Up Kit (Qiagen Inc., Valencia, CA). RNA samples (Control (pEV control), SPARC-overexpression and SPARC-underexpression D283 medulloblastoma cells) were processed at Ocean Ridge Bioscience (ORB, Palm Beach Gardens, FL, USA) for analysis using custom multi-species microarrays containing 1209 human mature probes miRNAs present in the miRBase version 16.0 database. An empty vehicle (pEV) control and SPARC overexpressed or SPARC underexpressed samples were assessed in biological triplicate. The sensitivity of microarray is such that it could detect as low as 20 amoles of synthetic miRNA being hybridized along with each sample. The microarrays were produced by Microarrays Inc. (Huntsville, Alabama), and made up of epoxide glass substrates that had been spotted in triplicate with each probe.

### miRNA sample processing

The LMW RNA samples were 3′-end labelled with Oyster-550 fluorescent dye using the Flash Taq RNA labelling Kit (Genisphere, Hatfield, PA). Labelled RNA samples were then hybridized to the miRNA microarrays according to the conditions outlined in the Flash Taq RNA labeling Kit protocol. The microarrays were scanned on an Axon Genepix 4000B scanner, and data was extracted from image using GenePix V4.1 software.

### Data pre-processing

Spot intensities were obtained for the 8816 features on each microarray by subtracting the medial local background from the median local foreground for each spot. The spot intensities and 95^th^ percentile of negative control spot was also calculated for each array. The spot intensities and 95^th^ percentile of negative controls (TPT95) were transformed by taking the Logarithm base 2 (indicated as Log2) of each value. The normalization factor (N) for each microarray was determined by achieving the 20 % trimmed mean of the human probe intensities that were detected one Log2 unit above TPT95 (TPT95 + 1) in all samples and with standard deviation of probe intensities among all samples less than 1.25. The Log2 transformed spot intensities for all 8816 features were normalized, by subtracting N from each spot intensity and scaled by adding the grand mean of N across all microarrays. The mean probe intensities for each of the 1209 human probes on each of the 20 arrays were then determined by averaging the triplicate spot intensities. Spots flagged as poor quality during data extraction were omitted prior to averaging. The 1209 human non- control Log2 transformed, normalized and averaged probe intensities were filtered to obtain a panel of 729 human miRNA probes showing probe intensity greater than one log2 unit above TPT95 (> TPT95 + 1) in at least 10 % of the samples.

### Quality control

Sensitivity of the microarray hybridization was confirmed by detection of hybridization signal for all 11 spikes that were added during isolation above the detection threshold level (labelling well above TPT95). The array also contains a set of specificity control probes complementary to three different miRNAs. Each specificity control includes a perfect match, signal mismatch, double mismatch and shuffled version of probes. Reproducibility of the arrays was determined by monitoring the hybridization intensity for the triplicate human spots on each array. Differential expression analysis

For statistical analysis, samples were binned in two groups: control and SPARC-overexpression or SPARC- underexpression. The Log2 transformed and normalized spot intensities for the 623 detectable were examined for difference between the groups by at least 2-fold difference between the control and SPARC-overexpression or SPARC-underexpression. A total 27 differentially expressed probes exhibited at-least two-fold difference between the control and SPARC-overexpression and/or SPARC-underexpression samples.

### Hierarchical clustering of miRNA array data

Data for the total 729 detectable human probes were clustered using Cluster 3.0 Software [74]. Genes were median-centered prior to hierarchical clustering. Hierarchical clustering was conducted using Centered Correlation as the similarity metric and Average Linkage as the clustering method. Intensity scale is shown in arbitrary units.

### Prediction of miRNA targets

To predict mRNA targets, a total 27 differentially expressed miRNAs which were identified in our analysis by looking at the at least 2-fold change difference between the control and SPARC-overexpression or SPARC-underexpression samples. These 27 miRNAs were uploaded into the Ingenuity Pathway Analysis (IPA) web-based tool (Ingenuity Systems, Redwood City, CA). Putative miRNA-mRNA relationships were identified using the IPA microRNA Target Filter, based on a knowledgebase of predicted and experimentally observed relationships. This database included interactions captured by Ingenuity Systems curators, as well as interactions from two external databases namely TarBase and miRecords. Furthermore, TarBase is a database that represents a comprehensive collection of miRNA targets with experimental support from published research articles. The interaction in TarBase are curated from both disease and non-disease related studies that use method to increase or decrease the expression of a particular miRNA of interest and evaluate its downstream transcriptional effects [75]. miRecords is a database of both experimentally validated miRNA targets that were captured through the compilation of findings from 2,705 studies, to date [76]. Moreover, the majority of the information in miRecords is collected from low-throughput experiments that also involve altering the expression levels of particular miRNAs in animal based experimental studies [76]. We stringently selected for only the experimentally observed miRNA-mRNA relationships, and the resulting target gene list was analyzed for functional network and biological pathway analysis using the PANTHER web tool (http://www.pantherdb.org/) [77]. The list of target genes was compared to a reference list to statistically determine over- or under-representation of PANTHER classification categories. *p-values* were calculated with Bonferroni correction test and *p < 0.05* was considered to estimate if a specific PANTHER category was over-or under- represented in a statistically significant way.

### Network analysis

The biologically network analyses were performed using IPA software. These mRNA targets with their respective miRNAs overlaid onto a global interaction network. Networks containing miRNA signaling where algorithmically evaluated by a modified Fisher’s exact test [78]. Biological functions and disease signatures within the constructed networks were then identified. Overrepresented functions/diseases were defined as those that contain more mRNA targets than expected by chance, as calculated by the right-tailed Fisher’s exact test with a *p value < 0.05* considered as a statistically significant.

### Data mining using the Oncomine Database for six miRNAs predicted gene targets

The expression pattern of six miRNAs predicted gene targets in desmoplastic medulloblastoma versus normal control samples were identified from Oncomine Database (www.oncomine.org/ Compendia Bioscience, Ann Arbor, MI, USA) [39, 40]. Oncomine is an online database that contains manually curated microarray and gene copy number expression data from various studies related to cancer. Currently, Oncomine Database contains data from more than 18,000 cancer gene expression microarray data from over 500 cancer types including human tumor samples and human cancer cell lines [39, 40]. The Oncomine platform allows us to compare gene expression across multiple cancer studies to identify genes that are differentially over or under expressed in different cancer types. Herein, we mined the microarray-gene expression data of human desmoplastic medulloblastoma cancer type samples versus normal control samples to identify six miRNAs predicted gene targets of SPARC overexpressed medulloblastoma cell samples versus control cell samples.

### miRNA qRT-PCR validation of six differentially expressed miRNAs in medulloblastoma

We tested the expression of levels of six miRNAs (has-miR-125b-1*, has-miR-146a-5p, has-miR-181a-5p, has-miR-204-5p, has-miR-219-5p and has-miR-509-3p) which were found to be differentially regulated with SPARC expression in medulloblastoma cells. Total RNA was extracted from D283 cells transfected with pSPARC or pEV using a miRNeasy Mini Plus kit (Qiagen, Valencia, CA) with QIAzol Lysis Reagent (Qiagen, Valencia, CA) for cell lysis. The RNA isolation procedure was accomplished using manufacture’s instruction. 1 μg of total RNA was used as a template for reverse transcription reaction using the miScript II Reverse Transcription kit (Qiagen, Valencia, CA), accordingly to the manufacturer’s instructions using 5X miScript HiFlex Buffer, which enables conversion of mature as well as precursor miRNAs. qRT-PCR was performed in 25 μl reaction using the miScript SYBR Green PCR kit (Qiagen, Valencia, CA), miRNA forward primers (primer sequences are presented in Table 1), and the reference U6 housekeeping miRNA in combination with the miScript Universal reverse primer (supplied with miScript SYBR Green PCR kit) for the cDNA templates. qRT-PCR reactions were performed in triplicate and independently repeated at least twice with no template control on 7500HT Fast Real-Time PCR System (Applied Biosystems, Carlsbad, CA, USA). PCRs were processed through an initial denaturation at 95°C for 15 min and by 40 cycles of 3-step PCR, including 15 sec of denaturation at 94°C, a 30 sec annealing phase at 55°C and an elongation phase at 70°C for 34 sec. The resulting RT-PCR cycle times were normalized against the U6 housekeeping miRNA, and fold change (FC) values of miRNA expression was calculated using the ΔΔCt method. Fold changes compared to controls were calculated by 2^−ΔΔCt^ after normalizing to U6, a reference gene. Statistical significance of the difference in miRNA expression levels between the empty vehicle (pEV) control and SPARC-overexpression (pSPARC) was calculated by unpaired *t*-tests in Graphpad Prism 5 Software (Graphpad Software Inc., San Diego, CA).

### Quantitative reverse-transcription polymerase chain reaction (qRT-PCR)

Total RNA was extracted from D283 mock or pEV control and pSPARC cells using TRIzol® solution (Invitrogen, Carlsbad, CA) as per standard protocol. For cDNA synthesis total RNA (1 μg) of was reverse transcribed with high-capacity cDNA reverse transcription kit (Applied Biosystems, Foster City, CA), and subsequently used to amplify SPARC, p53 and p21 using iTaq^TM^ Universal SYBR® Green Supermix kit (Bio-Rad Laboratories, Hercules, CA). GAPDH was used as a housekeeping reference gene in this analysis. qRT-PCR reaction mix was performed using the CFX96^TM^ Real- Time PCR System (Bio-Rad Laboratories, Hercules, CA) with following primers: SPARC forward, 5′-ATC TAA ATC CAC TCC TTC CAC AG-3′ and reverse, 5′-CAC CGT TAA TGT ATT CAC TTA AAT C-3′; p53 forward, 5′-ACA CGC TTC CCT GGA TTG-3′ and reverse, 5′- TCG ACG CTA GGA TCT GAC TG-3′; p21 forward, 5′-GAG GCC GGG ATG AGT TGG GAG GAG-3′ and reverse, 5′-CAG CCG GCG TTT GGA GTG GTA GAA- 3′ and GAPDH forward, 5′-GAA GGT GAA GGT CGG AGT C-3′ and reverse, 5′-GAA GAT GGT GAT GGG ATT TC-3′. Fold changes between the pSPARC and the control group (mock or pEV) were calculated using delta- delta cycle threshold (ΔΔCt) values and normalized with *GAPDH* as a housekeeping gene. Statistical significance of the transcript levels between pSPARC versus the control group was calculated using an unpaired *t-*test in Graphpad Prism 5 Software (Graphpad Software Inc., San Diego, CA).

## SUPPLEMENTARY MATERIALS TABLES


